# Assessment of Caregiver-Targeted Interventions for Use of Motor Vehicle Passenger Safety Systems for Children: A Systematic Review and Meta-analysis

**DOI:** 10.1001/jamanetworkopen.2019.14180

**Published:** 2019-10-30

**Authors:** Emma Sartin, Tyler R. Bell, Catherine C. McDonald, Jessica Hafetz Mirman

**Affiliations:** 1 Department of Psychology, University of Alabama at Birmingham; 2 Center for Injury Research and Prevention, Children’s Hospital of Philadelphia, Philadelphia, Pennsylvania; 3 College of Nursing, Pennsylvania State University, Philadelphia; 4 School of Nursing, University of Pennsylvania, Philadelphia; 5 The School of Health in Social Science, University of Edinburgh, Edinburgh, Scotland; 6 The Scottish Collaboration for Health Research and Policy, University of Edinburgh, Edinburgh, Scotland

## Abstract

**Importance:**

Caregiver-targeted interventions to improve the use of child restraint systems (CRS) in motor vehicles are common and heterogeneous in their implementation. The effectiveness of these interventions is unknown.

**Objectives:**

To quantify the effects of caregiver-targeted interventions using meta-analytic methods, assess the quality of published studies, and assess for publication bias.

**Data Sources:**

PubMed and PsychINFO (January 1, 2004, to April 1, 2019) were searched for English-language studies using a list of search terms. The search and screening process was completed between May 25, 2018, and April 1, 2019.

**Study Selection:**

Studies met inclusion criteria if they included a caregiver-targeted intervention that focused on increasing CRS use for children (age, ≤9 years) and report the use of CRS before and after the intervention.

**Data Extraction and Syntheses:**

Cochrane and PRISMA guidelines were used for the meta-analysis and risk-of-bias review. Information was extracted on intervention type, setting, implementation, and attributes of the study independently between 2 coders. Data were pooled from independent samples, with 1 outcome measure from each intervention implementation or study.

**Main Outcomes and Measures:**

This study was an exploratory random-effects meta-analysis. Unadjusted odds ratios were calculated using the sample size and the observed number of children in incorrect or correct restraints in motor vehicles before and after the intervention to determine the odds of incorrect CRS use after completing an intervention. Setting, measurement method, randomization, use of vouchers, and types of restraint were tested as moderators. A funnel plot was used to assess for publication bias.

**Results:**

Of 1240 potential articles, 51 were deemed eligible for screening and 10 (8238 participants total) were included in the meta-analysis. Caregiver-targeted interventions were associated with a reduction in the number of children not riding in a CRS (odds ratio, 0.51; 95% CI, 0.36-0.71; *P* < .001). Variance in the averaged effect size was driven by self-report methods (when removed from analyses, *I*^2^ = 61.8%; *R*^2^ change = 26.3; *P* = .02) and hospital settings (when removed from analyses, *I*^2^ = 70.7%; *R*^2^ change = 17.4; *P* = .002). Risk of bias was high in most studies; however, there was low evidence for publication bias.

**Conclusions and Relevance:**

In this meta-analysis, caregiver-targeted interventions were associated with a reduction in the number of children not riding in a CRS in motor vehicles; however, the methodological rigor of intervention studies should be enhanced.

## Key Points

**Question:**

Are caregiver-targeted child occupant protection interventions associated with changes in the use of motor vehicle child restraint systems?

**Findings:**

In this systematic review and meta-analysis of 10 studies comprising 8238 participants, caregiver-targeted interventions were found to be associated with a decrease in the number of children not riding in a motor vehicle restraint system. Most intervention studies included may be at a high risk for bias, but there was no observable evidence of publication bias.

**Meaning:**

Caregiver-targeted interventions are a promising method of promoting protection of children in motor vehicles, although more rigorous studies are needed to identify specific characteristics of the interventions that are successful.

## Teaser

This systematic review and meta-analysis evaluates whether caregiver-targeted interventions are associated with changes in the use of child restraint systems in motor vehicles.

## Introduction

There have been great strides in child occupant protection during the last several decades, with deaths to child occupants of motor vehicles reduced from 10.5 to 3.2 per 100 000 children between 1970 and 2007.^1^ This success is largely due to the introduction of child restraint systems (CRS) in conjunction with child occupant protection laws. To reduce injuries and deaths due to motor vehicle collisions, CRS couple the child with the vehicle to distribute the force of a collision across the strongest parts of a child’s body and prevent ejection.^2^ The American Academy of Pediatrics and the National Highway Traffic Safety Administration guidelines for CRS use are based on a child’s progress on major developmental milestones and his or her weight and height. Broadly, this guidance stipulates that an infant sit in a rear-facing car seat or convertible seat, transition to a forward-facing 5-point harness seat, and then progress to a belt-positioning booster seat in context of manufacturer guidelines specific to each CRS.^3^

All 50 states have some form of child restraint laws, which vary regarding requirements, penalties, and enforcement. Despite the benefits of correct CRS use, rates of children traveling inappropriately restrained, riding in an adult seatbelt, or unrestrained remain high.^4,5^ Although evidence suggests that restraint laws are efficacious, they are macrolevel interventions^6^ and can only indirectly influence caregivers’ behavior. Microlevel interventions, or those that target caregivers directly, can be useful for complementing policy-level efforts and are routine fixtures in many public health departments and pediatric care settings.^7^ However, intervention approaches vary (eg, clinical vs community setting), as do the study designs (eg, self-report vs observation) and it is not clear what approach to targeting caregivers might be most effective. Therefore, the current study is a meta-analysis of caregiver-targeted interventions designed to promote CRS use. Risk of bias assessments were completed for each study, along with an assessment of possible publication bias.

The meta-analysis was implemented following the Cochrane Review Guidelines, which recommend using only interventions that have a control group. Additional criteria for included articles were as follows: (1) must be a caregiver-targeted intervention, (2) with a focus on increasing CRS use for children old enough to use a booster seat or younger (ie, not seat belt use for adolescents), and (3) must report CRS use before and after the intervention (observed or self-report). For these analyses, we restricted the outcome to increase in CRS use, and not overall appropriateness of CRS use (eg, we did not include outcome measures of errors in the analysis).

## Methods

**Figure 1.**
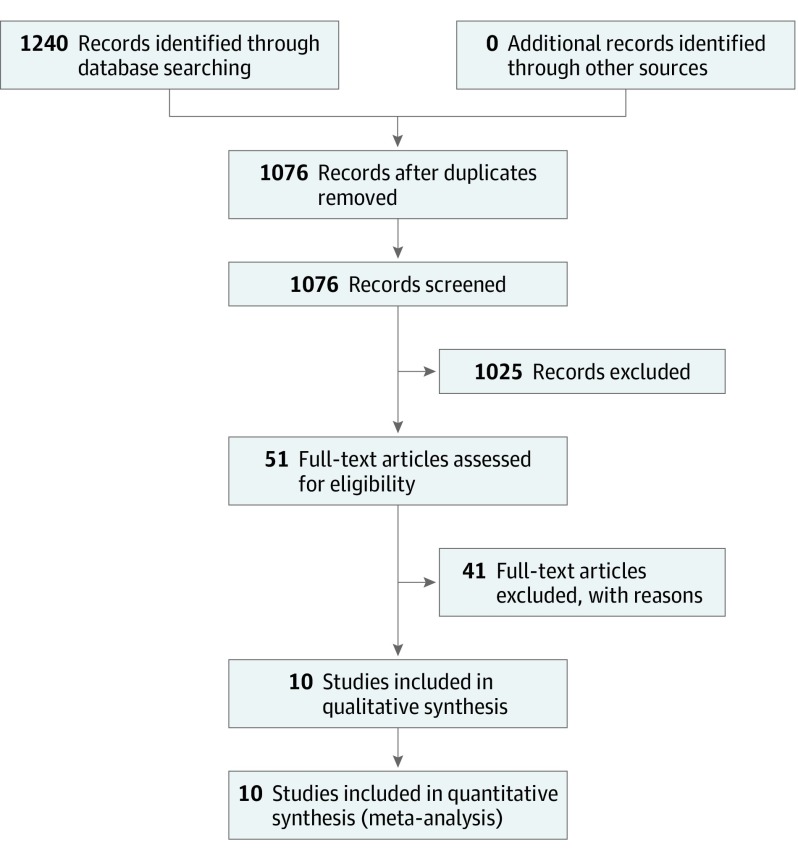
Flowchart of Search and Screening Process Search and screening process completed May 2018 to April 2019.

The search and screening process are represented in Figure 1. Literature searches were conducted in PsycINFO and PubMed and restricted to studies published between January 1, 2004, and April 1, 2019. The search and screening process was completed between May 25, 2018, and April 1, 2019. Three searches were completed by 3 reviewers in each database, using the following search strings: (search 1) *child passenger safety,* (search 2) *booster seat use,* and (search 3) *car seat use.* In PsycINFO, searches were restricted to peer-reviewed articles with an age group criterion of childhood (birth to 12 years). In PubMed, searches were restricted to human species and an age group criterion of birth to 18 years. Any identified articles that were literature reviews or previous meta-analyses of CRS interventions were examined to ensure that all articles possible were found and included in this analysis. All search result references were downloaded and entered into an Excel (Microsoft Corp) file that included detailed information about the study’s aims, participant population, findings, and a “relevancy” score determined by the reviewer (1 = extremely relevant, 2 = maybe relevant, and 3 = not at all relevant). A total of 1240 abstracts were found; however, after the initial review of abstracts for relevancy and duplicates, only 51 articles were determined to be relevant and were fully screened for inclusion in the analyses. This study followed the Preferred Reporting Items for Systematic Reviews and Meta-analyses (PRISMA) reporting guideline.^8^

After reviewing the full remaining studies, the 2 independent reviewers (E.S. and T.R.B.; 95% agreement; κ = 79.7%) agreed to remove 41 additional studies because of at least 1 of the following reasons: the study focused on the pre-post effects of legislation (n = 17), the study measured only an outcome other than an increase in CRS use (ie, parental knowledge or total number of errors in installations; n = 2), the study did not have a control group (ie, were pre-post designs; n = 15), the study focused on pedestrian training (n = 1), the study reported data in a way that odds ratios (ORs) could not be computed or in units that could not be compared with other studies (eg, mean percentage daily use over time, only collecting data for a control group after intervention, or overall restraint use for all children surveyed including those too large for booster seats; n = 3), or the study targeted a population that was not caregivers (ie, children or health care professionals; n = 2). One study was removed from the final analysis because it was reported to be an underpowered pilot study (ie, direct effects of the intervention to control group should not be compared).^9^ This process resulted in 10 studies for the final analyses.

### Data Extraction

Data were pooled from independent samples to meet the assumption of independence in our statistical analyses. Therefore, we used only 1 outcome measure from each intervention in each of the analyses. One intervention was reported in 2 different articles. Buckle Up Safely was implemented at the same time with different racial/ethnic target populations and reported separately (Buckle Up Safely in child care centers was reported by Keay et al^10^ [focused on the general population] and by Hunter et al^11^ [focused on the Aboriginal and Torres Strait Islander population]). In addition, a study by St Louis et al^12^ contains intervention efforts that targeted separate populations (lower socioeconomic status populations or Hispanic populations). These interventions were evaluated based on the success and rigor of their reported implementation and results (compliance measures, process evaluations, and effect sizes), and based on these criteria 1 set of data was included to represent each intervention (Keay et al^10^ and the Hispanic population from St Louis et al^12^).

After the final studies were identified, unadjusted ORs were calculated using the sample size and the observed number of children riding in CRS in motor vehicles before and after the intervention to determine the odds of incorrect child safety seat use after completing an intervention. Smaller values indicate a decreased likelihood of having a child unrestrained (ie, a smaller OR indicates the wanted effect of the intervention). For these analyses, we did not use data that only reported specific errors in CRS use or data reporting the accuracy of the installation-only data on whether the child was in a CRS (yes or no answer).

Risk of bias was assessed according to the Cochrane guidelines, with classifications of low, uncertain, or high across multiple domains.^13^ The domains included random sequence generation, allocation concealment, blinding of participants and personnel, blinding of outcome assessment, incomplete outcome data, selective reporting, group similarity at baseline, compliance, intent-to-treat analysis, and timing of outcome assessments. If a study did not report on a method, it was classified as an uncertain risk.

### Statistical Analysis

First, a random-effects meta-analysis using the DerSimonian and Laird method was conducted in OpenMeta^14,15^ to determine the association of caregiver-targeted interventions with use of CRS. This analysis provides a pooled OR effect size and 95% CI while estimating the between-study variance (*I*^2^). Next, setting of the intervention (community, hospital, virtual or using a technological platform, and child care center), how the study measured increase in CRS use (self-report or observation), randomization (yes or no), whether the intervention provided participants with seats (yes or no), and the target CRS use based on age of child population (booster seat only vs harnessed seats only or any type of restraint) were tested individually as moderators of the association between CRS use and each intervention. This analysis provides adjusted pooled log (OR) estimates that determine group-specific effect sizes (subgroup analyses show the unadjusted pooled ORs in eTable 1 in the Supplement). For significant moderators, exclusion analyses estimated variance explained (*R*^2^) by specific moderator subgroups. Last, a funnel plot visually inspected the possible extent to which publication bias influenced obtained results. As a sensitivity analysis, meta-analyses were conducted without studies outside the funnel to determine potential effects of publication bias on main results. All *P* values were from 2-sided tests and results were deemed statistically significant at *P* < .05.

## Results

Characteristics of the 10 identified studies including the target child age, total sample size, length of intervention, design, how the study measured CRS use, setting, mode of delivery, and intervention and control group components are presented in the Table.^10,12,16,17,18,19,20,21,22,23^ Interventions were delivered in 4 settings: communities (n = 3), hospitals (n = 3), child care centers (n = 2), and through a computer kiosk or a mobile phone app (n = 2). One of the hospital-based interventions and all of the interventions that were delivered in child care centers or in the community used observational methods to report changes in CRS use (n = 6), while the rest of the studies reported changes in CRS use with caregiver self-report (n = 4).

**Table.** Characteristics of Included Studies

Source	Target Child Age	Total Sample Size, No.	Length of Intervention	Design	Measure	Setting	Delivery	Components	
								Intervention	Control
Aitken et al,^16^ 2013	4-8 y	761	4-6 wk	NR	Obs	Community	Community intervention and marketing campaign	Community capacity building, awareness, education, and check-up events	Comparison communities that received information only
Gielen et al,^17^ 2007	4-66 mo	759	SD	R	SR	Virtual technology	Computer kiosk	Personalized education report and recommendations for appropriate car seat	Information and education about other health topics
Gielen et al,^18^ 2018	4-7 y	742	SD	R	SR	Virtual technology	Mobile phone app	Tailored information about CRS use	Information about fire safety
Gittelman et al,^19^ 2006	4-7 y	147	SD	R	SR	Hospital	In person	Education; education and free seat and installation	Standard discharge instructions
Istre et al,^20^ 2011	0-8 y	3554	30 mo	NR	Obs	Community	Community intervention	Awareness program, educational classes, voucher system, and check-up events	Comparison communities with no intervention or exposure
Keay et al,^10^ 2012	3-5 y	689	~7 mo	CR	Obs	Child care centers	In person	Education for staff of centers, educational DVD, information pack, information session, and vouchers	Matched centers with no intervention
Liu et al,^21^ 2016	Newborn	88	SD	R	SR	Hospital	In person	Education; education and free seat	Informational pamphlet about nutrition and food safety
St Louis et al,^12^ 2008	4-8 y	364	15 mo	NR	Obs	Community	Community intervention	Fitting station, education, and media	Comparison communities with no exposure or intervention
Tessier,^22^ 2010	Newborn	124	SD	R	Obs	Hospital	In person	Education and seat with installation demonstration	Education and seat with manual
Thoreson et al,^23^ 2009	4-8 y	1010	18 mo	CR	Obs	Child care centers	In person	Child center staff training, caregiver education event, resource kits, free booster seats, and promotions	Comparison centers with no intervention

Abbreviations: CR, cluster randomized; CRS, child restraint systems; NR, nonrandomized; R, randomized; Obs, observational; SD, single dose; SR, self-report.

### Meta-analysis

**Figure 2.**
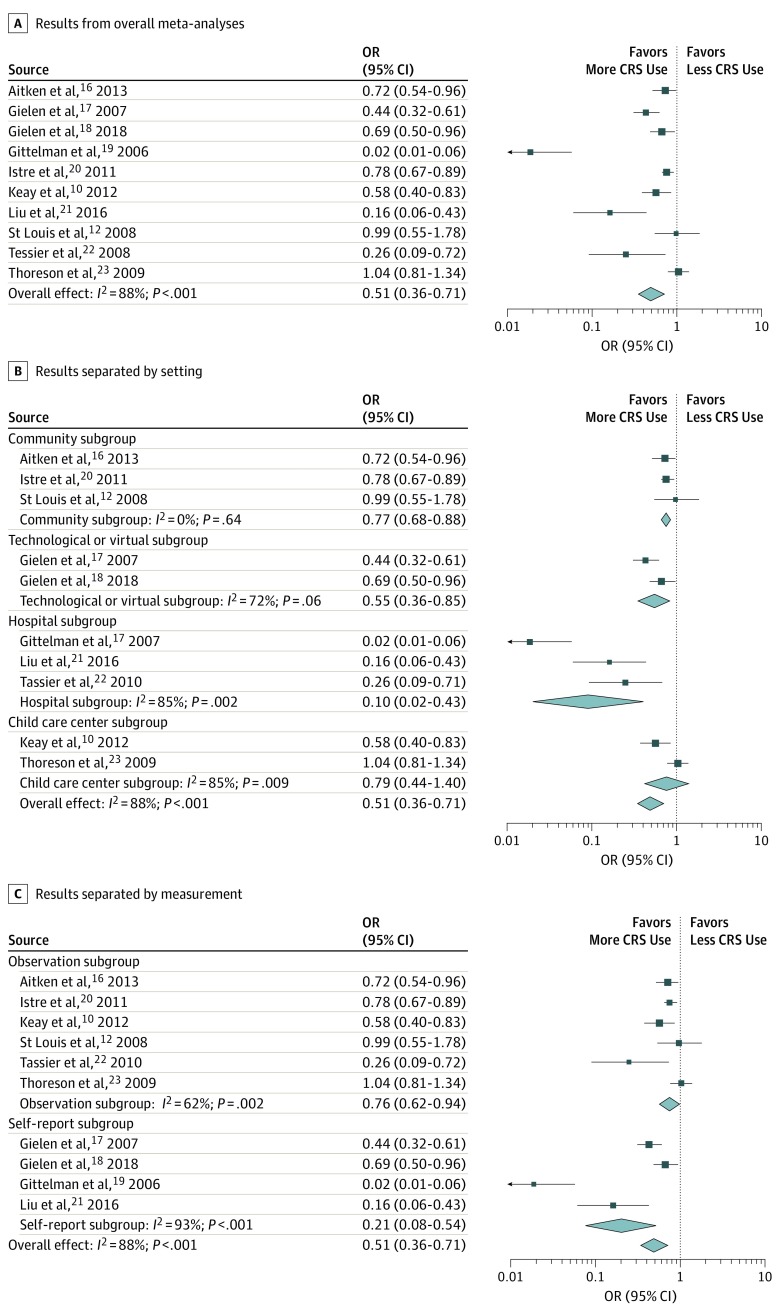
Forest Plots of Results From Meta-analyses A, Results from overall meta-analysis. B, Results separated by setting. C, Results separated by measurement. Diamonds represent 95% CIs. Squares indicate the effect sizes for each study, with lines representing each study’s 95% CI. Odds ratios (ORs) less than 1 indicate decreased odds of a child not being restrained in a child restraint system (CRS), while ORs greater than 1 indicate increased odds of a child not being restrained in a CRS.

The meta-analysis revealed that caregiver-targeted interventions were effective in decreasing the number of children not sitting in appropriate CRS (OR, 0.51; 95% CI, 0.36-0.70; *P* < .001; Figure 2), although there was high heterogeneity across studies (*I*^2^ = 88.1%; *P* < .001). The only moderator analyses that were statistically significant were the effects of setting and measurement method, ie, interventions in hospital settings (adjusted mean [SE] log (OR), −2.28 [0.31]; 95% CI, −2.90 to −1.67; *P* < .001) and studies that used self-report to test changes in CRS behaviors (adjusted mean [SE] log (OR), −1.52 [0.42]; 95% CI, −2.33 to −0.70; *P* = .03). Next, because these variables had high SEs, we tested the difference in heterogeneity of the analysis if studies with these characteristics were removed (eTable 1 in the Supplement). When studies with hospital settings were removed, there was a significant decrease in heterogeneity of the analysis (*I*^2^ = 70.7%; *R*^2^ change = 17.4; *P* = .002), with studies conducted in hospital settings accounting for 17.4% of the between-study variance explained by setting. When studies using self-report methods were removed, there was also a significant decrease in heterogeneity of the analysis (*I*^2^ = 61.8%; *R*^2^ change = 26.3; *P* = .02), with self-report methods accounting for 26.3% of the between-study variance explained by measurement. All but 1 of the interventions in the hospital setting also used self-report.

### Risk of Bias

Each study’s risk-of-bias classification across domains is presented in eTable 2 in the Supplement. All the studies were deemed to have low risk in selective reporting or timing of outcome assessments. Five of the studies used a low-risk qualifying randomization method for group allocation, while the other 5 studies used either a high-risk or unclear method. Only 3 studies received low-risk classifications for allocation concealment. Eight of the 10 studies were deemed to be unclear or high risk for outcome blinding, meaning most data were potentially collected or analyzed by personnel who knew each participant’s group assignment. Most studies, even those with multiple intervention sites, did not report compliance estimates.

### Publication Bias

**Figure 3.**
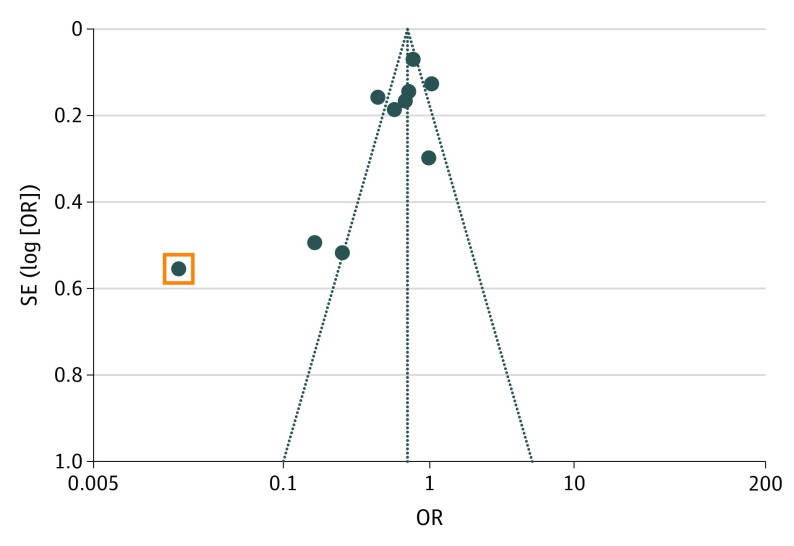
Funnel Plot for Risk of Publication Bias Publication bias is assessed by observing if the dots on the graph are symmetrical or not. Asymmetrical graphs suggest bias. The square represents the study by Gittelman et al,^19^ which was tested and found to not significantly change the overall results. OR indicates odds ratio.

Last, a funnel plot was constructed to evaluate for publication bias (Figure 3). There was no evidence for notable publication bias that might confound results; studies were proportionally centered around the aggregate estimate. Only 1 study fell outside the expected limits and might have introduced bias.^19^ However, when the article was removed from the analysis, the results were similar (OR, 0.74; 95% CI, 0.67-0.81; *P* < .001; *I*^2^ = 76%; *P* < .001).

## Discussion

The purpose of this meta-analysis was to undertake an in-depth and systematic evaluation of the published evidence and to determine the effects of caregiver-targeted interventions to increase CRS use. Ten studies were included based on the intervention target, the type of restraint use promoted (booster seat or overall CRS use), the type of data reported (CRS use, with data in format such that unadjusted ORs could be calculated), and interventions that included a control group. The results of our analysis support the use of caregiver-targeted interventions, as they appear to reduce the number of children not riding in a CRS.

Although this finding does support the use of caregiver-targeted interventions, there were high amounts of heterogeneity across the studies. The structure and methods of the interventions varied, as did the components of the interventions. Moderation analyses found that this heterogeneity was driven largely by interventions that were conducted in a hospital setting (3 interventions) and that used self-report methods (4 interventions). According to the forest plots, these studies typically had larger effects, but also larger amounts of error. More important, self-report methods of measuring CRS practices may overestimate actual CRS use.^10^

Another potential factor that was not used as a moderator in this study but could be affecting heterogeneity is the participants’ demographic information (race/ethnicity, age, and socioeconomic status). These variables were not used as moderators in our analysis because this information was not always reported in the included studies. For example, several of the interventions were community-based; therefore, individual race/ethnicity information was not always reported for the experimental groups. This is an important factor to consider, as previous research has suggested that racial/ethnic minority populations’ behaviors (including CRS use) are typically more difficult to influence through interventions and legislative efforts, or racial/ethnic minority populations react differently than do white populations to interventions and legislative efforts. For example, one community-based study included in this analysis targeted a specific ethnic group (Hispanic) and a low-income population: the reported results were different in each group.^12^ Another variable to be considered in the future is the length of the intervention. Community interventions in this analysis may have lasted more than 2 years, while hospital interventions were single-dose educational sessions. Differences in dose and time of implementation may have affected the influence of hospital settings on the overall heterogeneity, although we posit that this was likely related to the high likelihood of these interventions using self-report methods.

More important, few studies had low risks of bias in most categories assessed. Efforts need to be taken to reduce the amount of bias in implementing CRS interventions. It is difficult to make conclusions about the effects of an intervention when outcome variables are not collected objectively, or when necessary information is not reported. Previous reviews that focus on efforts to improve booster seat use have found that, overall, the included studies lacked scientific rigor in many categories considered.^6,11^ Based on our assessment, this finding has not changed in more recent efforts, especially when considering measures of compliance and methods of allocation. Finally, our funnel plot revealed that there does not seem to be a publication bias in this topic, which is a positive finding for the field overall.

### Limitations

These meta-analyses have several important limitations. One caveat to our findings is that there was a large amount of heterogeneity in the observed effects. Although it is an important finding in itself, this heterogeneity may have influenced the results by skewing the effects away from the true effect. Second, an effort was made to include as many studies as possible that focused on caregiver-targeted interventions; however, because of the way that some studies presented their data, we could not calculate unadjusted ORs, which may have influenced the results of this analysis by excluding data that were relevant to this analysis, specifically because of the small number of studies included. Third, although we visually inspected funnel plots, publication bias remains possible.

## Conclusions

Future efforts should test participant demographic variables, specifically race/ethnicity, as moderators on the effects of the interventions. This testing is important, as previous work in this area has highlighted the need for tailored interventions for minority populations. The heterogeneity in effects across methods in this analysis highlights a need to determine if a single-dose educational session can be as effective as the more costly and time-intensive community efforts. Child restraint systems are effective at protecting child occupants, but many families are still not using them appropriately or effectively. These results suggest that caregiver-targeted interventions are effective at improving rates of CRS use; however, future work examining efforts to improve CRS-related outcomes and efforts with more rigorous intervention evaluations are needed.

## Notes

Supplement. **eTable 1.** Subgroup Comparisons of Setting and Measurement, With Results After Excluding Significant Variables of Heterogeneity **eTable 2.** Risk of Bias Classifications
