# Supplementary material for: Assessment of Caregiver-Targeted Interventions for Use of Motor Vehicle Passenger Safety Systems for Children: A Systematic Review and Meta-analysis
Source: JAMA Netw Open. 2019 Oct 30;2(10):e1914180. doi: 10.1001/jamanetworkopen.2019.14180 (PMC6824219; doi:10.1001/jamanetworkopen.2019.14180)
Supplement: Supplement. — eTable 1. Subgroup Comparisons of Setting and Measurement, With Results After Excluding Significant Variables of Heterogeneity eTable 2. Risk of Bias Classifications [file jamanetwopen-2-e1914180-s001.pdf]

Supplementary Online Content

Sartin E, Bell TR, McDonald CC, Mirman JH. Assessment of caregiver-targeted interventions for use of motor vehicle passenger safety systems for children: a systematic review and meta-analysis. *JAMA Netw Open.* 2019;2(10):e1914180. doi:10.1001/jamanetworkopen.2019.14180

eTable 1. Subgroup Comparisons of Setting and Measurement, With Results After Excluding Significant Variables of Heterogeneity
eTable 2. Risk of Bias Classifications

This supplementary material has been provided by the authors to give readers additional information about their work.

© 2019 Sartin E et al. *JAMA Network Open.*

eTable 1. Subgroup Comparisons of Setting and Measurement, With Results After Excluding Significant Variables of Heterogeneity

Variable	Group	k	Estimate	95% CI	SE	*P* Value	Z	I^2	*P* Value
Setting									
	Community	3	0.774	0.68, 0.88	0.063	<.001	-4.048	0%	<.001
	Hospital	3	0.095	0.02, 0.43	0.77	0.002	-3.055	84.61%	0.002
	Virtual/Technology	2	0.552	0.36, 0.85	0.219	0.007	-2.72	71.75%	0.06
	Child Care Center	2	0.786	0.44, 1.4	0.294	0.415	-0.816	85.24%	0.009
Measurement									
	Observation	6	0.762	0.62, 0.94	0.109	0.013	-2.495	61.82%	0.022
	Self-report	4	0.207	0.08, 0.54	0.492	0.001	-3.201	92.91%	<.001

Model	OR (95% CI)	*P* Value	T2	Q	I^2	*P* Value	R^2 Change (%)
Original Model	0.507 (0.36, 0.71)	<.001	0.211	75.596	88.1	<0.001	-
Excluding Hospital	0.718 (0.59, 0.88)	0.001	0.048	20.475	70.7	0.002	17.4
Excluding Self-Report	0.762 (0.62, 0.94)	0.013	0.037	13.096	61.8	0.02	26.3

© 2019 Sartin E et al. *JAMA Network Open.*

eTable 2. Risk of Bias Classifications

Study	Random Sequence Allocation	Allocation Concealment	Blinding of Participants & Personnel	Blinding of Outcome Assessment	Incomplete outcome data	Group Similarity at Baseline	Compliance	Intent-to-Treat Analysis	Analysis Notes
Aitken et al., 2013	High risk-intervention sites self-selected/opted in	High risk-no allocation concealment	High risk-no blinding	High risk-No blinding of outcome assessment	Unclear-Random individuals from community were observed pre-and post, however there was a 25% reduction in the number of participants attributed to normal rates of attrition in community baseball leagues	High risk-participating groups were different across sociodemographic variables and baseline CRS use	High risk-authors report unanticipated factors altered planned sequence of intervention in some of the communities	Unclear-since this is a community based study it is hard to determine if individuals were completely separate/not exposed to any of the campaign	For this analysis, the odds ratio of the intervention's effect on CRS use was computed across all intervention sites

© 2019 Sartin E et al. *JAMA Network Open.*

Study	Random Sequence Allocation	Allocation Concealment	Blinding of Participants & Personnel	Blinding of Outcome Assessment	Incomplete outcome data	Group Similarity at Baseline	Compliance	Intent-to-Treat Analysis	Analysis Notes
Gielen et al., 2007	Low risk- A random number generation program assigned the participants to groups	Low risk- The participants completed the intervention through a virtual platform after the computer randomly assigned them to a group	Low risk- intervention was delivered via computer kiosk, so participant and personnel were not aware of which treatment the participant did or did not receive	Low risk- Interviewers collecting outcome data were blinded to participant's group	Low risk- attrition was less than 20% for both groups, and both groups experienced about equal amount	Low risk- No differences between groups on sociodemographic variables, reason for E.D. visits, or caregiver anxiety levels	Low risk- single dose individual sessions delivered via computer generated report	Low risk- all participants were analyzed in the group to which they were randomly assigned	For this analysis, we compared the high-exposure intervention group to the control group
Gielen et al., 2018	Low risk- block randomization by computer	Low risk- randomized by computer	Low risk- Surveys collected via smart phone app	Unclear- Authors do not report if the data was analyzed blindly	Low risk- authors adjusted models for propensity to remain in the study to account for differences in attrition based on sociodemographic variables	Low risk- No differences between groups in sociodemographic variables or across study sites	Unclear- intervention delivered via smart phone app, no information about compliance across participant provided	Low risk- All participants were analyzed in the group to which they were randomly assigned	N/A

© 2019 Sartin E et al. *JAMA Network Open.*

Study	Random Sequence Allocation	Allocation Concealment	Blinding of Participants & Personnel	Blinding of Outcome Assessment	Incomplete outcome data	Group Similarity at Baseline	Compliance	Intent-to-Treat Analysis	Analysis Notes
Gittelman et al., 2006	Low risk- random number table used to assign participants to groups	High risk- participant were assigned using an open random allocation schedule	Low risk- outcome not likely to be influenced by lack of blinding, as main difference between groups was whether the family received a free CRS	Unclear- not reported	Unclear- authors report high attrition rates across groups, however no differences in who completed follow up and who did not, and no differences across groups in who did not complete follow up	Low risk- groups appear similar at baseline	Unclear- single dose intervention, no information about compliance across individuals	Low risk- all participants analyzed in the group to which they were randomly assigned	Originally three groups: control group, education only, and education + free seat. For this analysis, we combined control and education only group to compare to education + free seat group.

© 2019 Sartin E et al. *JAMA Network Open.*

Study	Random Sequence Allocation	Allocation Concealment	Blinding of Participants & Personnel	Blinding of Outcome Assessment	Incomplete outcome data	Group Similarity at Baseline	Compliance	Intent-to-Treat Analysis	Analysis Notes
Istre et al., 2011	High risk- no randomization	High risk- no allocation concealment	High risk- no blinding	High risk- no blinding of outcome assessment	Unclear risk- random individuals in communities observed for pre-post observations	High risk- populations in communities seem different, although not statistically tested in analyses	Unclear- no measures of compliance across communities reported	Unclear- cannot confirm that no individuals in comparison community were not exposed to campaign	For this analysis we used unadjusted frequencies of child restraint use post-intervention, however authors note this was calculated to be in compliance with state law or not, which may include older children in seat belts only

© 2019 Sartin E et al. *JAMA Network Open.*

Study	Random Sequence Allocation	Allocation Concealment	Blinding of Participants & Personnel	Blinding of Outcome Assessment	Incomplete outcome data	Group Similarity at Baseline	Compliance	Intent-to-Treat Analysis	Analysis Notes
Keay et al., 2012	Unclear risk- authors report centers were approached in a random order and then randomly allocated into intervention or control, but no information given on the method of randomization	Unclear- no information given about method of randomization	High risk- centers and implementing staff aware of which group they were in based on design	High risk- observer were technically blinded to each center's allocation, however it is possible that they could tell which center was an intervention center based on materials around the campus	High risk- differences in caregivers who agreed to be observed vs. those who did not agree to be observed- only 52% of families approached agreed to be observed	Low risk- centers approached based on similar demographic characteristics and size	Unclear risk- compliance across centers is not reported	Unclear risk- data were collected within a specific time frame to avoid children and families in control groups being exposed to intervention, however cannot confirm that no individuals in comparison community were not exposed to campaign	N/A

© 2019 Sartin E et al. *JAMA Network Open.*

Study	Random Sequence Allocation	Allocation Concealment	Blinding of Participants & Personnel	Blinding of Outcome Assessment	Incomplete outcome data	Group Similarity at Baseline	Compliance	Intent-to-Treat Analysis	Analysis Notes
Liu et al., 2016	High risk- control group assigned based on being a patient at a specific hospital. Participants randomized by odd or even month of study	High risk- personnel aware of allocation because of odd vs. even month method	High risk- personnel were not blinded	Unclear- it is not reported if the personnel who collected outcome measured were blinded	High risk- attrition in the study was about 20%, and authors report that this may have influenced the outcome	Low risk- groups were balanced on sociodemographic measures at baseline	Unclear- single dose session, not reported across individuals	Low risk- all individuals analyzed in group to which they were randomized	For this analysis, we used education + free car seats compared to control group (education only group was excluded)
St. Louis et al., 2008	High risk- no randomization	High risk- no allocation concealment	High risk- no blinding	High risk- no blinding of outcome assessment	Unclear risk- Random individuals from community were observed pre-and post, no individual follow-up	High risk- comparison communities similar on US census information, but had higher rates of restraint use at baseline than intervention group	High risk- compliance across sites was an issue in the larger study, however this appeared to be less of an issue in the Hispanic only community	Unclear risk- cannot confirm that no individuals in comparison community were not exposed to campaign	For this analysis, we only used data from the Hispanic intervention community and its comparison community since the low-income community had issues in implementation

© 2019 Sartin E et al. *JAMA Network Open.*

Study	Random Sequence Allocation	Allocation Concealment	Blinding of Participants & Personnel	Blinding of Outcome Assessment	Incomplete outcome data	Group Similarity at Baseline	Compliance	Intent-to-Treat Analysis	Analysis Notes
Tessier 2010	Low risk- a random number was chosen to assign groups	Unclear risk- not enough information to determine if personnel could predict allocation	Low risk- there was no blinding, however it is unlikely this affected the results	Unclear risk- it is not reported if the personnel who collected outcome measured were blinded	Low risk- attrition was less than 20% for both control and intervention groups	Unclear risk- group specific information is not presented, just the overall sample	Unclear risk- single dose session, not reported across individuals	Low risk- all participants were analyzed in the group to which they were randomized	N/A

© 2019 Sartin E et al. *JAMA Network Open.*

Study	Random Sequence Allocation	Allocation Concealment	Blinding of Participants & Personnel	Blinding of Outcome Assessment	Incomplete outcome data	Group Similarity at Baseline	Compliance	Intent-to-Treat Analysis	Analysis Notes
Thoreson et al., 2009	Low risk- personnel involved in recruitment randomly assigned centers by minimization	Low risk- personnel who randomized centers not involved in recruitment	High risk- no blinding	Low risk- observers of outcome were blinded	Unclear risk- including vehicles not contacted or screened for observation, about 25% of eligible drivers participated in post-intervention surveys. 1 center in the intervention group and 3 centers in the control group were lost to follow up	Low risk- Because of minimization, groups were balanced on most sociodemographic variables	High risk- compliance across centers was not reported, however the authors did mention that all participating centers were analyzed regardless of the extent to which they implemented the program	Unclear risk- This is a center-based study it is difficult to determine individual treatment effects or if the comparison center individuals had any exposure to the intervention	N/A

eTable 2 Notes: Assessments found that all studies had low risk of timing of outcome assessments and selective reporting, so those are not presented in the table to preserve space.

© 2019 Sartin E et al. *JAMA Network Open.*
